# Supplementary material for: Confirming the statistically significant superiority of tree-based machine learning algorithms over their counterparts for tabular data
Source: PLoS One. 2024 Apr 18;19(4):e0301541. doi: 10.1371/journal.pone.0301541 (PMC11025817; doi:10.1371/journal.pone.0301541)
Supplement: S1 Table — (DOCX) [file pone.0301541.s001.docx]

Supplementary Table 1: Paired-sample t-test results for the precision, recall and F1 score measures between tree-based and non-tree-based trained supervised machine learning algorithms for the datasets from disease prediction (66) and university-ranking contexts (50).

(a) Precision measure (Disease prediction context)

| **Test** | **Group details** | | **Mean** | | **N** | **t** | **Sig.** |
| --- | --- | --- | --- | --- | --- | --- | --- |
|  | *Tree-based* | *Non-tree-based* | *Mean 1* | *Mean 2* |  |  |  |
| 1 | Random forest | Support vector machine | 0.99588 | 0.88287 | 66 | 7.263 | <0.001 |
| 2 | Random forest | Logistic regression | 0.99588 | 0.86031 | 66 | 8.368 | <0.001 |
| 3 | Random forest | K-nearest neighbour | 0.99588 | 0.89788 | 66 | 8.600 | <0.001 |
| 4 | Decision tree | Support vector machine | 0.99592 | 0.88287 | 66 | 7.265 | <0.001 |
| 5 | Decision tree | Logistic regression | 0.99592 | 0.86031 | 66 | 8.369 | <0.001 |
| 6 | Decision tree | K-nearest neighbour | 0.99592 | 0.89788 | 66 | 8.602 | <0.001 |

(b) Recall measure (Disease prediction context)

| **Test** | **Group details** | | **Mean** | | **N** | **t** | **Sig.** |
| --- | --- | --- | --- | --- | --- | --- | --- |
|  | *Tree-based* | *Non-tree-based* | *Mean 1* | *Mean 2* |  |  |  |
| 1 | Random forest | Support vector machine | 0.99575 | 0.89766 | 66 | 8.093 | <0.001 |
| 2 | Random forest | Logistic regression | 0.99575 | 0.87265 | 66 | 8.703 | <0.001 |
| 3 | Random forest | K-nearest neighbour | 0.99575 | 0.89919 | 66 | 8.630 | <0.001 |
| 4 | Decision tree | Support vector machine | 0.99575 | 0.89766 | 66 | 8.093 | <0.001 |
| 5 | Decision tree | Logistic regression | 0.99575 | 0.87265 | 66 | 8.703 | <0.001 |
| 6 | Decision tree | K-nearest neighbour | 0.99575 | 0.89919 | 66 | 8.630 | <0.001 |

(c) F1 score measure (Disease prediction context)

| **Test** | **Group details** | | **Mean** | | **N** | **t** | **Sig.** |
| --- | --- | --- | --- | --- | --- | --- | --- |
|  | *Tree-based* | *Non-tree-based* | *Mean 1* | *Mean 2* |  |  |  |
| 1 | Random forest | Support vector machine | 0.99573 | 0.88081 | 66 | 7.659 | <0.001 |
| 2 | Random forest | Logistic regression | 0.99573 | 0.85666 | 66 | 8.607 | <0.001 |
| 3 | Random forest | K-nearest neighbour | 0.99573 | 0.89418 | 66 | 8.659 | <0.001 |
| 4 | Decision tree | Support vector machine | 0.99565 | 0.88081 | 66 | 7.661 | <0.001 |
| 5 | Decision tree | Logistic regression | 0.99565 | 0.85666 | 66 | 8.609 | <0.001 |
| 6 | Decision tree | K-nearest neighbour | 0.99565 | 0.89418 | 66 | 8.661 | <0.001 |

(d) Precision measure (University-ranking context)

| **Test** | **Group details** | | **Mean** | | **N** | **t** | **Sig.** |
| --- | --- | --- | --- | --- | --- | --- | --- |
|  | *Tree-based* | *Non-tree-based* | *Mean 1* | *Mean 2* |  |  |  |
| 1 | Random forest | Support vector machine | 1.00000 | 0.99191 | 50 | 5.152 | <0.001 |
| 2 | Random forest | Logistic regression | 1.00000 | 0.98651 | 50 | 6.122 | <0.001 |
| 3 | Random forest | K-nearest neighbour | 1.00000 | 0.97981 | 50 | 6.831 | <0.001 |
| 4 | Decision tree | Support vector machine | 1.00000 | 0.99191 | 50 | 5.152 | <0.001 |
| 5 | Decision tree | Logistic regression | 1.00000 | 0.98651 | 50 | 6.122 | <0.001 |
| 6 | Decision tree | K-nearest neighbour | 1.00000 | 0.97981 | 50 | 6.831 | <0.001 |

(e) Recall measure (University-ranking context)

| **Test** | **Group details** | | **Mean** | | **N** | **t** | **Sig.** |
| --- | --- | --- | --- | --- | --- | --- | --- |
|  | *Tree-based* | *Non-tree-based* | *Mean 1* | *Mean 2* |  |  |  |
| 1 | Random forest | Support vector machine | 1.00000 | 0.99189 | 50 | 5.171 | <0.001 |
| 2 | Random forest | Logistic regression | 1.00000 | 0.98652 | 50 | 6.105 | <0.001 |
| 3 | Random forest | K-nearest neighbour | 1.00000 | 0.97987 | 50 | 6.871 | <0.001 |
| 4 | Decision tree | Support vector machine | 1.00000 | 0.99189 | 50 | 5.171 | <0.001 |
| 5 | Decision tree | Logistic regression | 1.00000 | 0.98652 | 50 | 6.105 | <0.001 |
| 6 | Decision tree | K-nearest neighbour | 1.00000 | 0.97987 | 50 | 6.871 | <0.001 |

(f) F1 score measure (University-ranking context)

| **Test** | **Group details** | | **Mean** | | **N** | **t** | **Sig.** |
| --- | --- | --- | --- | --- | --- | --- | --- |
|  | *Tree-based* | *Non-tree-based* | *Mean 1* | *Mean 2* |  |  |  |
| 1 | Random forest | Support vector machine | 1.00000 | 0.99184 | 50 | 5.123 | <0.001 |
| 2 | Random forest | Logistic regression | 1.00000 | 0.98634 | 50 | 6.147 | <0.001 |
| 3 | Random forest | K-nearest neighbour | 1.00000 | 0.97960 | 50 | 6.823 | <0.001 |
| 4 | Decision tree | Support vector machine | 1.00000 | 0.99184 | 50 | 5.123 | <0.001 |
| 5 | Decision tree | Logistic regression | 1.00000 | 0.98634 | 50 | 6.147 | <0.001 |
| 6 | Decision tree | K-nearest neighbour | 1.00000 | 0.97960 | 50 | 6.823 | <0.001 |
