# Supplementary material for: Confirming the statistically significant superiority of tree-based machine learning algorithms over their counterparts for tabular data
Source: PLoS One. 2024 Apr 18;19(4):e0301541. doi: 10.1371/journal.pone.0301541 (PMC11025817; doi:10.1371/journal.pone.0301541)
Supplement: S2 Table — (DOCX) [file pone.0301541.s002.docx]

Supplementary Table 2: Dataset source information

| **ID** | **Dataset name** | **Source** | **Web link** |
| --- | --- | --- | --- |
| D1 | Heart Disease Dataset (Comprehensive) | Kaggle | Heart Disease Dataset (Comprehensive) \| Kaggle |
| D2 | Health care: Heart attack possibility | Kaggle | Health care: Heart attack possibility \| Kaggle |
| D3 | Heart Disease Dataset | Kaggle | https://www.kaggle.com/datasets/johnsmith88/heart-disease-dataset |
| D4 | Liver Disorders | UCI | https://archive.ics.uci.edu/ml/datasets/Liver+Disorders |
| D5 | Diabetes prediction system with KNN algorithm | Kaggle | https://www.kaggle.com/abdallamahgoub/diabetes |
| D6 | Bank Marketing (dataset -1) | UCI | https://archive.ics.uci.edu/dataset/222/bank+marketing |
| D7 | Bank Marketing (dataset -2) | UCI | https://archive.ics.uci.edu/dataset/222/bank+marketing |
| D8 | Mushroom | UCI | https://archive.ics.uci.edu/dataset/73/mushroom |
| D9 | Chronic Kidney Disease dataset | Kaggle | https://www.kaggle.com/datasets/mansoordaku/ckdisease |
| D10 | Breast Cancer prediction | Kaggle | https://www.kaggle.com/code/buddhiniw/breast-cancer-prediction |
| D11 | Stroke Prediction Dataset | Kaggle | https://www.kaggle.com/datasets/fedesoriano/stroke-prediction-dataset |
| D12 | Lung Cancer Prediction | Kaggle | https://www.kaggle.com/datasets/thedevastator/cancer-patients-and-air-pollution-a-new-link |
| D13 | Hepatitis | UCI | https://archive.ics.uci.edu/dataset/46/hepatitis |
| D14 | Ionosphere | UCI | https://archive.ics.uci.edu/dataset/52/ionosphere |
| D15 | Thyroid Disease | UCI | https://archive.ics.uci.edu/dataset/102/thyroid+disease |
| D16 | Heart Failure Prediction | Kaggle | https://www.kaggle.com/datasets/andrewmvd/heart-failure-clinical-data |
| D17 | Parkinson's | UCI | https://archive.ics.uci.edu/dataset/174/parkinsons |
| D18 | Indian Liver Patient Dataset | Kaggle | https://www.kaggle.com/datasets/jeevannagaraj/indian-liver-patient-dataset |
| D19 | COVID-19 Effect on Liver Cancer Prediction Dataset | Kaggle | https://www.kaggle.com/datasets/fedesoriano/covid19-effect-on-liver-cancer-prediction-dataset |
| D20 | Liver dataset | Kaggle | https://www.kaggle.com/datasets/vishnupriya99/liver-dataset |
| D21 | Specht Heart | UCI | https://archive.ics.uci.edu/dataset/95/spect+heart |
| D22 | Early-stage diabetes risk prediction dataset | UCI | https://archive.ics.uci.edu/dataset/529/early+stage+diabetes+risk+prediction+dataset |
| D23 | Diabetic Retinopathy Debrecen Data Set | UCI | https://archive.ics.uci.edu/dataset/329/diabetic+retinopathy+debrecen+data+set |
| D24 | Breast Cancer Coimbra | UCI | https://archive.ics.uci.edu/dataset/451/breast+cancer+coimbra |
| D25 | Chronic Kidney Disease | Kaggle | https://www.kaggle.com/datasets/abhia1999/chronic-kidney-disease |
| D26 | Kidney Stone Dataset | Kaggle | https://www.kaggle.com/datasets/harshghadiya/kidneystone |
| D27 | Chess (King-Rook vs. King-Pawn) | UCI | https://archive.ics.uci.edu/dataset/23/chess+king+rook+vs+king |
| D28 | Echocardiogram | UCI | https://archive.ics.uci.edu/dataset/38/echocardiogram |
| D29 | Bladder Cancer Recurrence Dataset | Kaggle | https://www.kaggle.com/datasets/utkarshx27/bladder-cancer-recurrences?select=bladder2.csv |
| D30 | Adult | UCI | https://archive.ics.uci.edu/dataset/2/adult |
| D31 | Prostate Cancer | Kaggle | https://www.kaggle.com/datasets/sajidsaifi/prostate-cancer |
| D32 | Telco Customer Churn | Kaggle | https://www.kaggle.com/datasets/blastchar/telco-customer-churn |
| D33 | EEG Eye State | UCI | https://archive.ics.uci.edu/dataset/264/eeg+eye+state |
| D34 | Spam base | UCI | https://archive.ics.uci.edu/dataset/94/spambase |
| D35 | Credit Approval | UCI | https://archive.ics.uci.edu/dataset/27/credit+approval |
| D36 | Tic-Tac-Toe Endgame | UCI | https://archive.ics.uci.edu/dataset/101/tic+tac+toe+endgame |
| D37 | Steel Plates Faults | UCI | https://archive.ics.uci.edu/dataset/198/steel+plates+faults |
| D38 | Online shoppers Purchasing Intention dataset | UCI | https://archive.ics.uci.edu/dataset/468/online+shoppers+purchasing+intention+dataset |
| D39 | fertility | UCI | https://archive.ics.uci.edu/dataset/244/fertility |
| D40 | IPL 2022 Match dataset | Kaggle | https://www.kaggle.com/datasets/vora1011/ipl-2022-match-dataset?select=IPL_Matches_2022.csv |
| D41 | League of Legends Diamond ranked games | Kaggle | https://www.kaggle.com/datasets/bobbyscience/league-of-legends-diamond-ranked-games-10-min |
| D42 | Airline customer Holiday Booking dataset | Kaggle | https://www.kaggle.com/datasets/manishkumar7432698/airline-passangers-booking-data |
| D43 | Student Marks | Kaggle | https://www.kaggle.com/datasets/shub99/student-marks |
| D44 | Student_2nd_Year_Result | Kaggle | https://www.kaggle.com/datasets/aqeebhussain/student-2nd-year-result |
| D45 | Student mat pass or fail | Kaggle | https://www.kaggle.com/datasets/ouline/student-mat-pass-or-fail |
| D46 | Real Breast Cancer Data | Kaggle | https://www.kaggle.com/datasets/amandam1/breastcancerdataset |
| D47 | Breast Cancer Dataset | Kaggle | https://www.kaggle.com/datasets/utkarshx27/breast-cancer-dataset-used-royston-and-altman |
| D48 | Lung Cancer Dataset | Kaggle | https://www.kaggle.com/datasets/yusufdede/lung-cancer-dataset |
| D49 | Air pressure system failures in Scania trucks | Kaggle | https://www.kaggle.com/datasets/uciml/aps-failure-at-scania-trucks-data-set?select=aps_failure_test_set.csv |
| D50 | Predict Term Deposit | Kaggle | https://www.kaggle.com/datasets/aslanahmedov/predict-term-deposit |
| D51 | Darwin | UCI | https://archive.ics.uci.edu/dataset/732/darwin |
| D52 | Cervical Cancer (Risk Factors) | UCI | https://archive.ics.uci.edu/dataset/383/cervical+cancer+risk+factors |
| D53 | Breast Cancer Wisconsin (Original) | UCI | https://archive.ics.uci.edu/dataset/15/breast+cancer+wisconsin+original |
| D54 | Breast Cancer Wisconsin (Original) | UCI | https://archive.ics.uci.edu/dataset/15/breast+cancer+wisconsin+original |
| D55 | League of Legends Diamond Ranked Games | Kaggle | https://www.kaggle.com/datasets/bobbyscience/league-of-legends-diamond-ranked-games-10-min |
| D56 | Chess game dataset | Kaggle | https://www.kaggle.com/datasets/datasnaek/chess |
| D57 | Games of Thrones | Kaggle | https://www.kaggle.com/datasets/mylesoneill/game-of-thrones |
| D58 | EEG Brainwave Dataset: Feeling Emotions | Kaggle | https://www.kaggle.com/datasets/birdy654/eeg-brainwave-dataset-feeling-emotions |
| D59 | Red Wine Quality | Kaggle | https://www.kaggle.com/datasets/uciml/red-wine-quality-cortez-et-al-2009 |
| D60 | white wine quality | Kaggle | https://www.kaggle.com/datasets/piyushagni5/white-wine-quality/code |
| D61 | Breast Cancer Prediction Dataset | Kaggle | https://www.kaggle.com/datasets/merishnasuwal/breast-cancer-prediction-dataset |
| D62 | Thyroid Disease | UCI | https://archive.ics.uci.edu/dataset/102/thyroid+disease |
| D63 | sick-euthyroid | UCI | https://archive.ics.uci.edu/dataset/102/thyroid+disease |
| D64 | Ann-test | UCI | https://archive.ics.uci.edu/dataset/102/thyroid+disease |
| D65 | Ann-train | UCI | https://archive.ics.uci.edu/dataset/102/thyroid+disease |
| D66 | Hypothyroid | UCI | https://archive.ics.uci.edu/dataset/102/thyroid+disease |
| D67 | New-thyroid | UCI | https://archive.ics.uci.edu/dataset/102/thyroid+disease |
| D68 | Lung cancer | Kaggle | https://www.kaggle.com/datasets/nancyalaswad90/lung-cancer |
| D69 | Cancer Patients Data | Kaggle | https://www.kaggle.com/datasets/rishidamarla/cancer-patients-data |
| D70 | Labor Relations | UCI | https://archive.ics.uci.edu/dataset/56/labor+relations |
| D71 | Glioma Grading Clinical and Mutation Features Dataset | UCI | https://archive.ics.uci.edu/dataset/759/glioma+grading+clinical+and+mutation+features+dataset |
| D72 | Glioma Grading Clinical and Mutation Features Dataset | UCI | https://archive.ics.uci.edu/dataset/759/glioma+grading+clinical+and+mutation+features+dataset |
| D73 | Connect-4 | UCI | https://archive.ics.uci.edu/dataset/26/connect+4 |
| D74 | Post-Operative Patient | UCI | https://archive.ics.uci.edu/dataset/82/post+operative+patient |
| D75 | Predict FIFA 2018 Man of the Match | Kaggle | https://www.kaggle.com/datasets/mathan/fifa-2018-match-statistics |
| D76 | Dota 2 Matches | Kaggle | https://www.kaggle.com/datasets/devinanzelmo/dota-2-matches?select=match.csv |
| D77 | IPL Match Analysis | Kaggle | https://www.kaggle.com/code/sahib12/ipl-match-analysis/input?select=matches.csv |
| D78 | CS: GO Professional Matches | Kaggle | https://www.kaggle.com/datasets/mateusdmachado/csgo-professional-matches?select=results.csv |
| D79 | IPL 2008 to 2022 All Match Dataset | Kaggle | https://www.kaggle.com/datasets/vora1011/ipl-2008-to-2021-all-match-dataset?select=IPL_Matches_2008_2022.csv |
| D80 | Heart Rate Prediction to Monitor Stress Level | Kaggle | https://www.kaggle.com/datasets/vinayakshanawad/heart-rate-prediction-to-monitor-stress-level |
| D81 | Predictive Maintenance Dataset (AI4I 2020) | Kaggle | https://www.kaggle.com/datasets/stephanmatzka/predictive-maintenance-dataset-ai4i-2020 |
| D82 | Diabetes Dataset 2019 | Kaggle | https://www.kaggle.com/datasets/tigganeha4/diabetes-dataset-2019 |
| D83 | Paris Housing Classification | Kaggle | https://www.kaggle.com/datasets/mssmartypants/paris-housing-classification |
| D84 | Oranges vs. Grapefruit | Kaggle | https://www.kaggle.com/datasets/joshmcadams/oranges-vs-grapefruit |
| D85 | Synthetic Binary Classification Dataset! | Kaggle | https://www.kaggle.com/datasets/kylegraupe/simulation-generated-dataset-binary-class |
| D86 | Synthetic Binary Classification Dataset! | Kaggle | https://www.kaggle.com/datasets/kylegraupe/simulation-generated-dataset-binary-class |
| D87 | Personal Key Indicators of Heart Disease | Kaggle | https://www.kaggle.com/datasets/kamilpytlak/personal-key-indicators-of-heart-disease |
| D88 | Airline Passenger Satisfaction | Kaggle | https://www.kaggle.com/datasets/teejmahal20/airline-passenger-satisfaction |
| D89 | Naive Bayes classification data | Kaggle | https://www.kaggle.com/datasets/himanshunakrani/naive-bayes-classification-data |
| D90 | Crystal System Properties for Li-ion Batteries | Kaggle | https://www.kaggle.com/datasets/divyansh22/crystal-system-properties-for-liion-batteries |
| D91 | Fake Bills | Kaggle | https://www.kaggle.com/datasets/alexandrepetit881234/fake-bills |
| D92 | Logistic Regression on Heart Disease Dataset | Kaggle | https://www.kaggle.com/code/pritishmishra/logistic-regression-on-heart-disease-dataset/input |
| D93 | Air pressure system failures in Scania trucks | Kaggle | https://www.kaggle.com/datasets/uciml/aps-failure-at-scania-trucks-data-set?select=aps_failure_test_set.csv |
| D94 | Go Emotions: Google Emotions Dataset | Kaggle | https://www.kaggle.com/datasets/shivamb/go-emotions-google-emotions-dataset |
| D95 | Diabetes prediction dataset | Kaggle | https://www.kaggle.com/datasets/iammustafatz/diabetes-prediction-dataset |
| D96 | Credit Card Fraud Detection | Kaggle | https://www.kaggle.com/datasets/mlg-ulb/creditcardfraud |
| D97 | Cardiovascular disease dataset | kaggle | https://www.kaggle.com/datasets/sulianova/cardiovascular-disease-dataset |
| D98 | Starter: Diabetes 130 US hospitals | Kaggle | https://www.kaggle.com/code/fakhrul77/starter-diabetes-130-us-hospitals-for-4e0c2549-f |
| D99 | Heart Disease Dataset | Kaggle | https://www.kaggle.com/datasets/mirzahasnine/heart-disease-dataset |
| D100 | Spotify Popular East Asian Artists and Tracks | Kaggle | https://www.kaggle.com/datasets/crxxom/spotify-popular-east-asian-artists-and-tracks?select=east_asia_top_tracks.csv |
| D101 | Pagila (PostgreSQL Sample Database) | Kaggle | https://www.kaggle.com/datasets/kapturovalexander/pagila-postgresql-sample-database?select=customer.csv |
| D102 | PepsiCo products Details: 180+ products | Kaggle | https://www.kaggle.com/datasets/mauryansshivam/pepsico-products-details-180-products |
| D103 | Students' Academic Performance Dataset | Kaggle | https://www.kaggle.com/datasets/aljarah/xAPI-Edu-Data |
| D104 | Student Academic Performance Analysis | Kaggle | https://www.kaggle.com/code/bhartiprasad17/student-academic-performance-analysis/input |
| D105 | Predict students' dropout and academic success | Kaggle | https://www.kaggle.com/datasets/thedevastator/higher-education-predictors-of-student-retention |
| D106 | Impact of Electronic Gadgets on Students' Results | Kaggle | https://www.kaggle.com/datasets/nabilajahan/the-impact-of-electronic-gadget-uses |
| D107 | You're Hired! \|Analysis of Campus Recruitment Data | Kaggle | https://www.kaggle.com/code/benroshan/you-re-hired-analysis-on-campus-recruitment-data/input |
| D108 | Predicting End-Semester Performance | Kaggle | https://www.kaggle.com/datasets/akiwelekar/predictingese?select=AttendanceMarksSA.csv |
| D109 | Fitbits, field-tests, and grades | Kaggle | https://www.kaggle.com/datasets/aleespinosa/fitbits-fieldtests-and-grades |
| D110 | Student Time Management Performance Dataset | Kaggle | https://www.kaggle.com/datasets/xiaowenlimarketing/international-student-time-management?select=Sheet2.csv |
| D111 | Student Feedback Dataset | Kaggle | https://www.kaggle.com/datasets/brarajit18/student-feedback-dataset?select=finalDataset0.2.xlsx |
| D112 | Depression and Academic performance of students | Kaggle | https://www.kaggle.com/datasets/kanerudolph/depression-and-academic-performance-of-students |
| D113 | Academic ranking of world universities Analytics | Kaggle | https://www.kaggle.com/datasets/shivan118/world-university-rankings-analytics?select=nineteen_twenty_university_datasets.xlsx |
| D114 | Video Games | Kaggle | https://www.kaggle.com/datasets/mohamedhanyyy/video-games |
| D115 | Video Games Sales Dataset | Kaggle | https://www.kaggle.com/datasets/sidtwr/videogames-sales-dataset?select=XboxOne_GameSales.csv |
| D116 | Video Games Sales Dataset | Kaggle | https://www.kaggle.com/datasets/sidtwr/videogames-sales-dataset?select=XboxOne_GameSales.csv |
| D117 | Sacred Games | Kaggle | https://www.kaggle.com/datasets/zusmani/sacred-games |
| D118 | PC Games Sales | Kaggle | https://www.kaggle.com/datasets/khaiid/most-selling-pc-games |
| D119 | Popular Video Games 1980 - 2023 | Kaggle | https://www.kaggle.com/datasets/arnabchaki/popular-video-games-1980-2023 |
| D120 | Olympic Games 2021 Medals | Kaggle | https://www.kaggle.com/datasets/stefanzivanov/olympic-games-2021-medals?select=Tokyo+2021+dataset+v4.csv |
| D121 | Video Games Rating By 'ESRB' | Kaggle | https://www.kaggle.com/datasets/imohtn/video-games-rating-by-esrb?select=test_esrb.csv |
| D122 | Top Games on the Google Play Store | Kaggle | https://www.kaggle.com/datasets/dhruvildave/top-play-store-games |
| D123 | Steam Games | Kaggle | https://www.kaggle.com/datasets/thedevastator/get-your-game-on-metacritic-recommendations-and |
| D124 | PS4 Games | Kaggle | https://www.kaggle.com/datasets/ww1234/ps4-games |
| D125 | Suicide rates by countries | Kaggle | https://www.kaggle.com/datasets/zvr842/suicude-rates-by-countries |
| D126 | Ultimate University Ranking (CWUR: 2012-2013) | Kaggle | https://www.kaggle.com/datasets/erfansobhaei/ultimate-university-ranking |
| D127 | Ultimate University Ranking (CWUR: 2013-2014) | Kaggle | https://www.kaggle.com/datasets/erfansobhaei/ultimate-university-ranking |
| D128 | Ultimate University Ranking (CWUR: 2014-2015) | Kaggle | https://www.kaggle.com/datasets/erfansobhaei/ultimate-university-ranking |
| D129 | Ultimate University Ranking (CWUR: 2015-2016) | Kaggle | https://www.kaggle.com/datasets/erfansobhaei/ultimate-university-ranking |
| D130 | Ultimate University Ranking (CWUR: 2016-2017) | Kaggle | https://www.kaggle.com/datasets/erfansobhaei/ultimate-university-ranking |
| D131 | Ultimate University Ranking (CWUR: 2017-2018) | Kaggle | https://www.kaggle.com/datasets/erfansobhaei/ultimate-university-ranking |
| D132 | Ultimate University Ranking (CWUR: 2018-2019) | Kaggle | https://www.kaggle.com/datasets/erfansobhaei/ultimate-university-ranking |
| D133 | Ultimate University Ranking (CWUR: 2019-2020) | Kaggle | https://www.kaggle.com/datasets/erfansobhaei/ultimate-university-ranking |
| D134 | Ultimate University Ranking (CWUR: 2020-2021) | Kaggle | https://www.kaggle.com/datasets/erfansobhaei/ultimate-university-ranking |
| D135 | Ultimate University Ranking (CWUR: 2021-2022) | Kaggle | https://www.kaggle.com/datasets/erfansobhaei/ultimate-university-ranking |
| D136 | Ultimate University Ranking (CWUR: 2022-2023) | Kaggle | https://www.kaggle.com/datasets/erfansobhaei/ultimate-university-ranking |
| D137 | Ultimate University Ranking (GM: 2016) | Kaggle | https://www.kaggle.com/datasets/erfansobhaei/ultimate-university-ranking |
| D138 | Ultimate University Ranking (GM: 2017) | Kaggle | https://www.kaggle.com/datasets/erfansobhaei/ultimate-university-ranking |
| D139 | Ultimate University Ranking (GM: 2018) | Kaggle | https://www.kaggle.com/datasets/erfansobhaei/ultimate-university-ranking |
| D140 | Ultimate University Ranking (GM: 2019) | Kaggle | https://www.kaggle.com/datasets/erfansobhaei/ultimate-university-ranking |
| D141 | Ultimate University Ranking (GM: 2020) | Kaggle | https://www.kaggle.com/datasets/erfansobhaei/ultimate-university-ranking |
| D142 | Ultimate University Ranking (GM: 2021) | Kaggle | https://www.kaggle.com/datasets/erfansobhaei/ultimate-university-ranking |
| D143 | Ultimate University Ranking (GM: 2022) | Kaggle | https://www.kaggle.com/datasets/erfansobhaei/ultimate-university-ranking |
| D144 | Ultimate University Ranking (Webometric: 2012) | Kaggle | https://www.kaggle.com/datasets/erfansobhaei/ultimate-university-ranking |
| D145 | Ultimate University Ranking (Webometric: 2013) | Kaggle | https://www.kaggle.com/datasets/erfansobhaei/ultimate-university-ranking |
| D146 | Ultimate University Ranking (Webometric: 2014) | Kaggle | https://www.kaggle.com/datasets/erfansobhaei/ultimate-university-ranking |
| D147 | Ultimate University Ranking (Webometric: 2015) | Kaggle | https://www.kaggle.com/datasets/erfansobhaei/ultimate-university-ranking |
| D148 | Ultimate University Ranking (Webometric: 2016) | Kaggle | https://www.kaggle.com/datasets/erfansobhaei/ultimate-university-ranking |
| D149 | Ultimate University Ranking (Webometric: 2017) | Kaggle | https://www.kaggle.com/datasets/erfansobhaei/ultimate-university-ranking |
| D150 | Ultimate University Ranking (Webometric: 2018) | Kaggle | https://www.kaggle.com/datasets/erfansobhaei/ultimate-university-ranking |
| D151 | Ultimate University Ranking (Webometric: 2019) | Kaggle | https://www.kaggle.com/datasets/erfansobhaei/ultimate-university-ranking |
| D152 | Ultimate University Ranking (Webometric: 2020) | Kaggle | https://www.kaggle.com/datasets/erfansobhaei/ultimate-university-ranking |
| D153 | Ultimate University Ranking (Webometric: 2021) | Kaggle | https://www.kaggle.com/datasets/erfansobhaei/ultimate-university-ranking |
| D154 | Ultimate University Ranking (Webometric: 2022) | Kaggle | https://www.kaggle.com/datasets/erfansobhaei/ultimate-university-ranking |
| D155 | Ultimate University Ranking (Webometric: 2023) | Kaggle | https://www.kaggle.com/datasets/erfansobhaei/ultimate-university-ranking |
| D156 | Ultimate University Ranking (URAP: 2018-2019) | Kaggle | https://www.kaggle.com/datasets/erfansobhaei/ultimate-university-ranking |
| D157 | Ultimate University Ranking (URAP: 2019-2020) | Kaggle | https://www.kaggle.com/datasets/erfansobhaei/ultimate-university-ranking |
| D158 | Ultimate University Ranking (URAP: 2020-2021) | Kaggle | https://www.kaggle.com/datasets/erfansobhaei/ultimate-university-ranking |
| D159 | Ultimate University Ranking (URAP: 2021-2022) | Kaggle | https://www.kaggle.com/datasets/erfansobhaei/ultimate-university-ranking |
| D160 | Ultimate University Ranking ((URAP: 2022-2023) | Kaggle | https://www.kaggle.com/datasets/erfansobhaei/ultimate-university-ranking |
| D161 | Ultimate University Ranking (THE: 2011) | Kaggle | https://www.kaggle.com/datasets/erfansobhaei/ultimate-university-ranking |
| D162 | Ultimate University Ranking (THE: 2012) | Kaggle | https://www.kaggle.com/datasets/erfansobhaei/ultimate-university-ranking |
| D163 | Ultimate University Ranking (THE: 2013) | Kaggle | https://www.kaggle.com/datasets/erfansobhaei/ultimate-university-ranking |
| D164 | Ultimate University Ranking (THE: 2014) | Kaggle | https://www.kaggle.com/datasets/erfansobhaei/ultimate-university-ranking |
| D165 | Ultimate University Ranking (THE: 2015) | Kaggle | https://www.kaggle.com/datasets/erfansobhaei/ultimate-university-ranking |
| D166 | Ultimate University Ranking (THE: 2016) | Kaggle | https://www.kaggle.com/datasets/erfansobhaei/ultimate-university-ranking |
| D167 | Ultimate University Ranking (THE: 2017) | Kaggle | https://www.kaggle.com/datasets/erfansobhaei/ultimate-university-ranking |
| D168 | Ultimate University Ranking (THE: 2018) | Kaggle | https://www.kaggle.com/datasets/erfansobhaei/ultimate-university-ranking |
| D169 | Ultimate University Ranking (THE: 2019) | Kaggle | https://www.kaggle.com/datasets/erfansobhaei/ultimate-university-ranking |
| D170 | Ultimate University Ranking (THE: 2020) | Kaggle | https://www.kaggle.com/datasets/erfansobhaei/ultimate-university-ranking |
| D171 | Ultimate University Ranking (THE: 2021) | Kaggle | https://www.kaggle.com/datasets/erfansobhaei/ultimate-university-ranking |
| D172 | Ultimate University Ranking (THE: 2022) | Kaggle | https://www.kaggle.com/datasets/erfansobhaei/ultimate-university-ranking |
| D173 | Ultimate University Ranking (THE: 2023) | Kaggle | https://www.kaggle.com/datasets/erfansobhaei/ultimate-university-ranking |
| D174 | Ultimate University Ranking (QS: 2022) | Kaggle | https://www.kaggle.com/datasets/erfansobhaei/ultimate-university-ranking |
| D175 | Monk’s Problems (Monks-1) | UCI | https://archive.ics.uci.edu/dataset/70/monk+s+problems |
| D176 | Monk’s Problems (Monks-2) | UCI | https://archive.ics.uci.edu/dataset/70/monk+s+problems |
| D177 | Monk’s Problems (Monks-3) | UCI | https://archive.ics.uci.edu/dataset/70/monk+s+problems |
| D178 | Monk’s Problems (Monks-4) | UCI | https://archive.ics.uci.edu/dataset/70/monk+s+problems |
| D179 | Monk’s Problems (Monks-5) | UCI | https://archive.ics.uci.edu/dataset/70/monk+s+problems |
| D180 | Monk’s Problems (Monks-6) | UCI | https://archive.ics.uci.edu/dataset/70/monk+s+problems |
| D181 | HCV Data | UCI | https://archive.ics.uci.edu/dataset/571/hcv+data |
| D182 | Estimation of obesity levels based on eating habits and physical condition | UCI | https://archive.ics.uci.edu/dataset/544/estimation+of+obesity+levels+based+on+eating+habits+and+physical+condition |
| D183 | Drug consumption (quantified) | UCI | https://archive.ics.uci.edu/dataset/373/drug+consumption+quantified |
| D184 | Cardiotocography | UCI | https://archive.ics.uci.edu/dataset/193/cardiotocography |
| D185 | Toxicity | UCI | https://archive.ics.uci.edu/dataset/728/toxicity-2 |
| D186 | Toxicity | UCI | https://archive.ics.uci.edu/dataset/728/toxicity-2 |
| D187 | Blood Transfusion Service Center | UCI | https://archive.ics.uci.edu/dataset/176/blood+transfusion+service+center |
| D188 | South German Credit | UCI | https://archive.ics.uci.edu/dataset/522/south+german+credit |
| D189 | Mammographic Mass | UCI | https://archive.ics.uci.edu/dataset/161/mammographic+mass |
| D190 | Student Academics Performance | UCI | https://archive.ics.uci.edu/dataset/467/student+academics+performance |
| D191 | Obesity Classification Dataset | Kaggle | https://www.kaggle.com/datasets/sujithmandala/obesity-classification-dataset |
| D192 | Gender Classification Dataset | Kaggle | https://www.kaggle.com/datasets/elakiricoder/gender-classification-dataset |
| D193 | Credit Risk Classification Dataset | Kaggle | https://www.kaggle.com/datasets/praveengovi/credit-risk-classification-dataset |
| D194 | Happiness Classification Dataset | Kaggle | https://www.kaggle.com/datasets/priyanshusethi/happiness-classification-dataset/discussion |
| D195 | Credit Score Classification Dataset | Kaggle | https://www.kaggle.com/datasets/sujithmandala/credit-score-classification-dataset |
| D196 | Banking Dataset Classification | Kaggle | https://www.kaggle.com/datasets/rashmiranu/banking-dataset-classification |
| D197 | Star Dataset for Stellar Classification | Kaggle | https://www.kaggle.com/datasets/vinesmsuic/star-categorization-giants-and-dwarfs |
| D198 | Star Dataset for Stellar Classification | Kaggle | https://www.kaggle.com/datasets/vinesmsuic/star-categorization-giants-and-dwarfs |
| D199 | Easiest Diabetes Classification Dataset | Kaggle | https://www.kaggle.com/datasets/sujithmandala/easiest-diabetes-classification-dataset |
| D200 | Monkey-Pox PATIENTS Dataset | Kaggle | https://www.kaggle.com/datasets/muhammad4hmed/monkeypox-patients-dataset |
